# Herbivory of oil-exposed submerged aquatic vegetation *Ruppia maritima*

**DOI:** 10.1371/journal.pone.0208463

**Published:** 2018-12-05

**Authors:** Charles W. Martin, Erick M. Swenson

**Affiliations:** 1 University of Florida/Institute of Food and Agricultural Sciences Nature Coast Biological Station, Cedar Key, Florida, United States of America; 2 Department of Oceanography and Coastal Sciences, Louisiana State University, Baton Rouge, Louisiana, United States of America; Department of Agriculture and Water Resources, AUSTRALIA

## Abstract

Oil spills, such as the Deepwater Horizon spill in the Gulf of Mexico, have the potential to dramatically alter coastal food webs through a variety of mechanisms. While oil can have direct impacts on primary producers through toxicity and shading, it is also possible that more subtle, indirect changes to the interactions among organisms could alter energy flow through the ecosystem. Here, we present the results of a series of manipulative experiments to determine the impacts of oil exposure on herbivory of *Ruppia maritima*, one of the most common species of submerged vegetation found in the region impacted by the 2010 Deepwater Horizon oil spill. In previous experiments, *R*. *maritima* was grown in a range of manipulated sediment oil concentrations. Using plant tissue from this experiment, we analyzed the effects of oil on plant chemical composition and found that plant carbon:nitrogen ratio (C:N) was reduced by as much as 21% in plants exposed to higher concentrations of oil. Given that nitrogen plays a key role in herbivore preference patterns, we performed herbivory assays and found oil-contaminated plants were preferred by herbivores in choice trials, although subsequent no-choice experiments indicated herbivores consumed less oil-contaminated tissue. We hypothesize the reason for this is that more tissue of higher C:N content is needed to meet similar metabolic demands while avoiding the potentially negative impacts of feeding on contaminated tissues. These results indicate that substantial food web alterations may occur via enhanced consumption of oil-exposed plants and provides vital information necessary to assess the large-scale impact of oil on submerged macrophytes.

## Introduction

The explosion of the Deepwater Horizon drilling rig off the coast of Louisiana in the Gulf of Mexico (GOM) was a large-scale disaster that resulted in loss of human life and numerous environmental consequences, many of which are still under investigation. Almost 5 million barrels of unrefined Sweet Louisiana Crude oil was released over a 3-month period in 2010, making it the largest marine spill in US history [1, 2]. Despite numerous efforts to protect coastal waters from released oil, including application of dispersants to enhance microbial breakdown of oil, burning of offshore oil, opening of Mississippi River control structures to increase freshwater discharge, and placement of oil protection booms and cleanup crews in strategic locations [3], more than 1700 km of shoreline was impacted in the northern GOM [4]. Oil impacted a wide variety of critical habitats from coastal Louisiana to the Florida Panhandle, including emergent marshes, oyster reefs, mangroves, beaches, and beds of submerged vegetation [4–7].

The coastal wetlands impacted by the spill provide a variety of key ecosystem functions that enhance the productivity and resilience of the region. Among these numerous ecosystem services, coastal wetlands provide abundant food sources and refugia for juvenile organisms (including those of commercial and recreational importance). While our knowledge of oil’s impact to emergent grasses such as *Spartina alterniflora* and *Juncus roemerianus* (reviewed in [8]) is substantial, much less is known about the effects on submerged vegetation. Given the lack of traditional monitoring and turbid waters that persist in many northern GOM estuaries, impact assessments of submerged vegetation fall far behind those of other macrophytes with fewer published studies (but see [6, 7, 9]) despite their importance as a food resource and refuge [10–13].

A variety of submerged grasses persist in the estuaries of the northern GOM that were impacted by this spill [14, 15]. Among the most abundant and widely distributed is *Ruppia maritima* (herafter referred to as *Ruppia*), especially in coastal Louisiana [16–18]. *Ruppia* is highly tolerant to the rapid, and often seasonal, fluctuations in salinity and other environmental parameters that occur in estuaries. It also supports a diverse food web, including invertebrates, fishes, and birds [19–21]. Given the numerous organisms and productive food web supported by *Ruppia*, understanding the impact of oil on both the plant itself and the larger ecosystem is critical to conserving and managing estuarine resources. We know from previous work [6] that *Ruppia* is resilient to many of oil’s negative effects, with sustained growth and biomass, although subtle impacts to reproduction, root morphology, and erosion can occur.

In general, seagrass and submerged vegetation support a diverse food web directly by providing energy and a food source for organisms (through herbivory), and indirectly as a refuge from predators [20, 22–24]. To date, a number of studies have quantified the herbivory of (primarily) marine macrophytes through both field assessments [22, 25] and laboratory investigations [26]. Herbivory of *Ruppia*, however, has received comparatively less attention (but see [23]). Given the food web supported by *Ruppia* [20], a better understanding of the role of *Ruppia* as a food source, and the concomitant effects of oil contamination, are needed to construct more detailed food web models and habitat linkages, as well as risk assessments to disturbances such as oil spills.

Here, we assess the impacts of oil on herbivory of *Ruppia*. Using tissue from a previous experiment that exposed *Ruppia* to a range of oil concentrations, we performed feeding assays under controlled laboratory conditions to determine: 1) herbivore preference (choice) patterns for *Ruppia* grown in oil, and 2) the rate of foraging on oil-exposed *Ruppia*. Coupled with these investigations is an analysis to determine the chemical composition (C:N ratio) of plants as a potentially important covariate in these assessments. The overarching aim of this research was to determine the potential alterations to the trophic transfer from primary producer to herbivore that occurs via oil contamination.

## Methods

### Oil exposure and plant chemical composition

Previously, Martin et al. [6] reported on the growth, reproduction, and morphological characteristics of *Ruppia* grown under 4 oil concentrations: none (0 mL oil/L tank), low (0.26 mL oil/L tank volume), medium (0.53 mL oil/L tank volume), and high (1.05 mL oil/L tank volume) oil. Plants were grown in randomized, aerated mesocosms and harvested after 1 month. These concentrations were within the range found under coastal Louisiana field conditions [27]. Additional experimental details and results can be found in Martin et al. [6]. We utilized plant tissue from that experiment to test whether oil exposure affects herbivore preference and foraging patterns.

Available plant leaf material, likely the most susceptible and frequently grazed portion of the plant, was analyzed for chemical composition to determine the carbon:nitrogen (C:N) ratio, as C:N ratio has been found to be a strong determinant for herbivore preference [26]. Leaves were removed from the stem, dried to a constant weight at 60°C, ground to a powder using a mortar and pestle, and analyzed for C:N content using established methods [28, 29]. Ten replicates were conducted for each oil treatment.

### Herbivore assays

To test herbivore preference patterns, two experiments were conducted: 1) choice tests and 2) foraging rate tests. Each experiment was performed using one of two herbivores: grass shrimp (*Palaemonetes pugio*) and amphipods (*Gammarus mucronatus*), both abundant and recognized herbivores of submerged vegetation [23, 24]. Pre-treatment husbandry conditions were consistent among animals that had been collected near Lake Pontchartrain, LA (USA) and held under identical lab conditions. We randomized individuals to treatments further ensuring we did not bias results due to herbivore selection. All trials using grass shrimp were conducted in 3.8 L aquaria with 5 shrimp in each and amphipod trials were conducted in 0.5 L aquaria with 8 amphipods in each, both within the range of natural densities found in the field. All aquaria were aerated and environmental conditions held constant throughout the duration of the trial (salinity 5–7, temperature 23°C). While shrimp and amphipod trials were conducted at separate times, as were choice and foraging rate experiments, all oil treatments were performed simultaneously and completely randomized and used individuals of consistent size. All herbivores were starved for 24 hours prior to the trial and no mortality was noted during trials.

Using remaining ground tissue from the above chemical analyses, artificial leaves were constructed by pouring agar and ground tissue over window screening cut to simulate the size of a leaf. Specifically, deionized water and agar at a ratio of 25 mL:0.3 g agar was microwaved for approximately 1 minute, poured into a plastic sheet with wells cut to fit the window screen, and 50 mg of plant tissue placed homogenously in each well. These methods are similar to previous herbivory assays [26, 28, 30, 31]. After the agar cooled, each artificial leaf was weighed on a balance pre- and post-experiment (described below). The proportion loss of material was calculated and used for all comparisons. During each experiment, autogenic controls with no herbivores were run to ensure that artificial leaves did not lose mass over the trial period; we did not note any loss of mass, therefore we did not include herbivore controls in subsequent analyses because inclusion of this data (all 0%, and thus no variance) would likely lead to a failure to meet the assumption of homogeneity of variance among treatments.

#### Experiment 1: Choice tests

In choice tests, each herbivore was offered a choice between an artificial leaf from 2 of the previously mentioned oil treatments. Each pairwise comparison was tested: None vs Low, None vs Medium, None vs High, Low vs Medium, Low vs High, and Medium vs High. Shrimp or amphipods were allowed to feed for 24 hours before the trial ended and artificial leaves removed, patted dry with a paper towel, and reweighed. Each unique choice comparison was replicated 12 times.

#### Experiment 2: Foraging rate tests

In foraging rate tests, we determined the rate at which herbivores feed on oil-contaminated tissues in the aforementioned concentrations. We offered herbivores one leaf (i.e., no choice) from each treatment. In this experiment, shrimp were allowed to feed for 24 hours and each treatment replicated 12 times. Amphipods were also allowed to feed for 24 hours. However, due to little feeding the experiment was repeated over 48 hours. Trials using amphipods in this experiment were replicated 18 times.

### Statistical analyses

Prior to any analysis, assumptions of parametric statistics (normality and homogeneity of variance) were tested and transformed if necessary. Plant C:N ratio was analyzed using a one-way analysis of variance with Tukey’s post-hoc analysis to determine which treatments differed. Choice tests were analyzed using paired t-tests for each individual comparison. Foraging rate comparisons were made using a one-way analysis of variance, again with Tukey’s post hoc pairwise comparisons. In choice and foraging rate tests, each herbivore was tested separately. Results were considered significant at p < 0.05.

### Ethics statement

Animals and plants used in this study were collected from wild populations on public lands. No vertebrates or protected species were used or impacted during this study. All collections were made under Louisiana Department of Wildlife and Freshwater Fisheries permit number SCP152.

## Results

### Oil exposure and plant chemical composition

**Fig 1:**
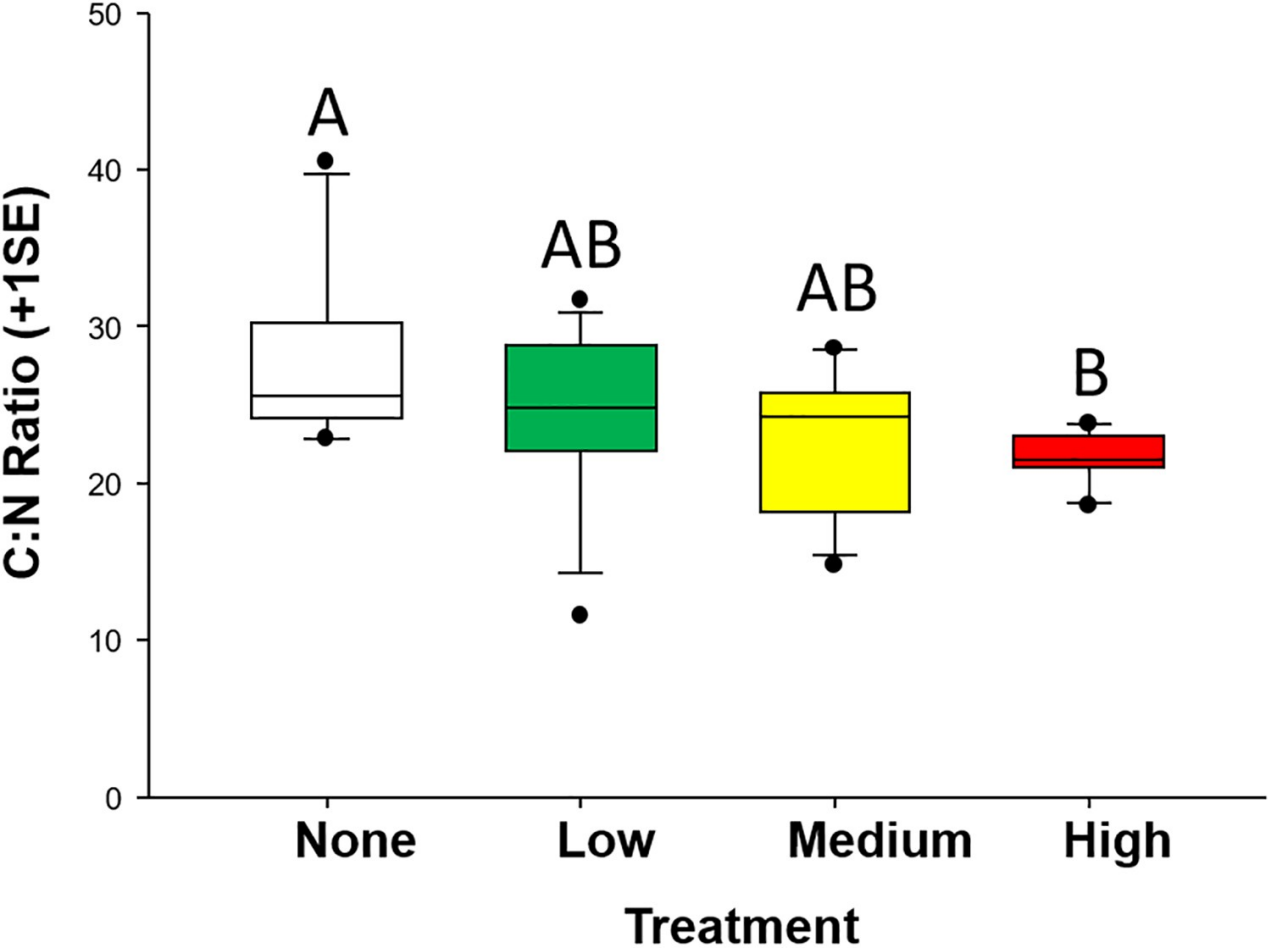
Carbon:nitrogen (C:N) ratio in plant tissues. C:N ratio in plants grown in no (white), low (green), medium (yellow), and high (red) oil-contaminated sediments. Different letters indicate statistically significant differences.

Plants grown in different oil concentrations had significantly different C:N ratios (F_3,36_ = 4.81, p = 0.006). A clear trend of decreasing C:N ratio with increasing oil exposure was detected (Fig 1), with post hoc comparisons indicating that no oil was significantly different than medium (p = 0.049) and high oil (p = 0.001). Average C:N ratio in no oil was approximately 27.5, and decreased to around 25.3, 23.0, and 21.7 in low, medium, and high oil, respectively.

### Herbivore assays

#### Experiment 1: Choice tests

**Fig 2:**
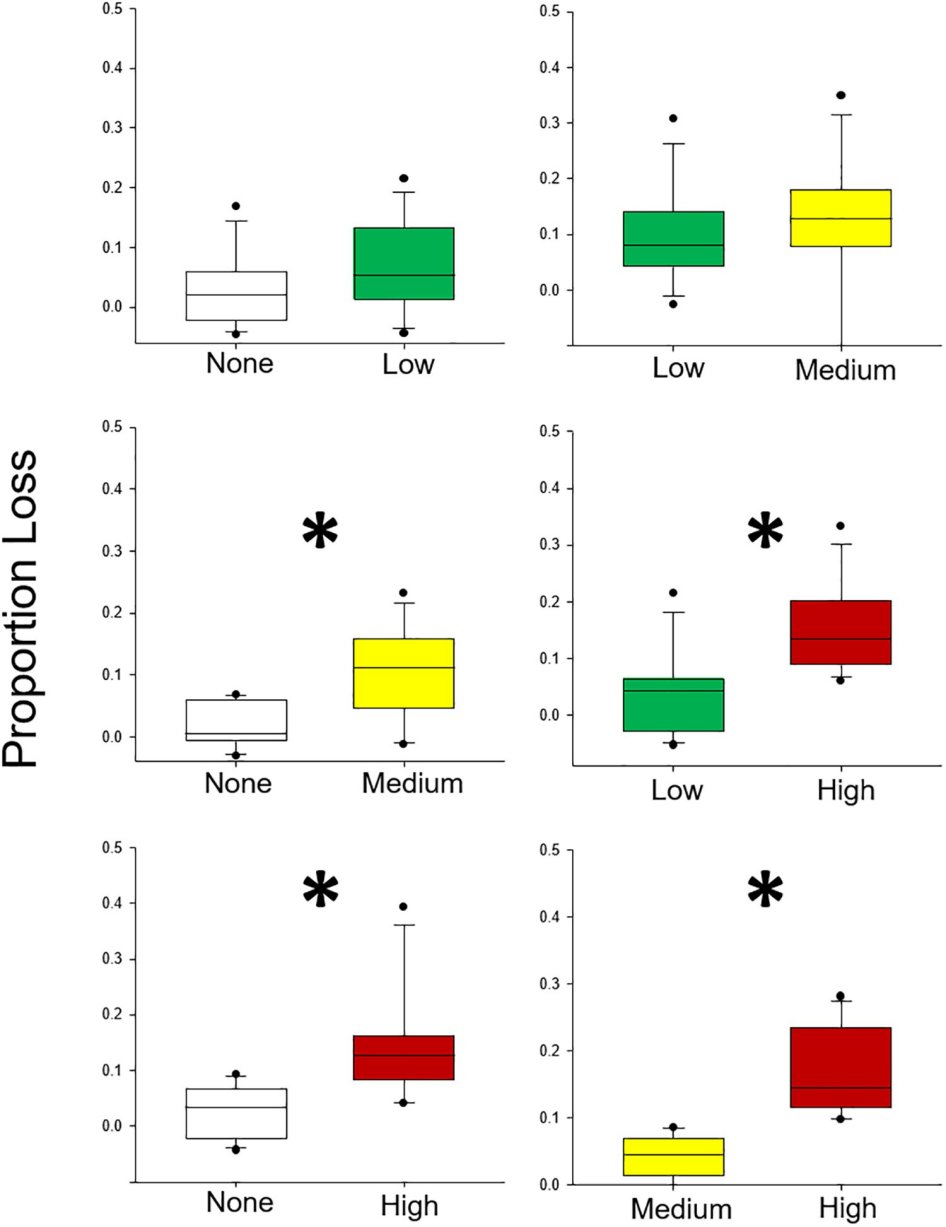
Results from Experiment 1: Grass shrimp. Paired choice experiments using grass shrimp (*P*. *pugio*) herbivores and plants grown in 4 oiled conditions: no (white), low (green), medium (yellow), and high (red). Asterisk indicates that a statistically significant difference exists.

Grass shrimp generally demonstrated preference patterns for grasses grown under higher oil concentrations (Table 1, Fig 2). Significant differences in foraging were detected for comparisons of None vs Medium, None vs High, Low vs High, and Medium vs High (Table 1). In each case, more material was consumed in the tissue with oil higher exposure (Fig 2). In general, shrimp consumed between 5 and 30% of material during trials, within the optimal range for foraging trials [32].

**Table 1:** Results from paired t-tests comparing herbivore preference patterns in Experiment 2 among plants with no (0 mL oil/L tank), low (0.26 mL oil/L tank volume), medium (0.53 mL oil/L tank volume), and high (1.05 mL oil/L tank volume) oil exposure. Significant results (p < 0.05) are shown in bold.

Herbivore	Comparison	T value	P-value
*Palaemonetes pugio*	None vs Low	-1.43	0.177
	**None vs Medium**	**-3.80**	**0.003**
	**None vs High**	**-3.06**	**< 0.001**
	Low vs Medium	0.56	0.586
	**Low vs High**	**-3.934**	**0.002**
	**Medium vs High**	**-6.88**	**< 0.001**
*Gammarus mucronatus*	None vs Low	1.97	0.074
	None vs Medium	-1.98	0.074
	**None vs High**	**2.99**	**0.012**
	Low vs Medium	0.27	0.791
	**Low vs High**	**-2.79**	**0.017**
	**Medium vs High**	**2.59**	**0.025**

**Fig 3:**
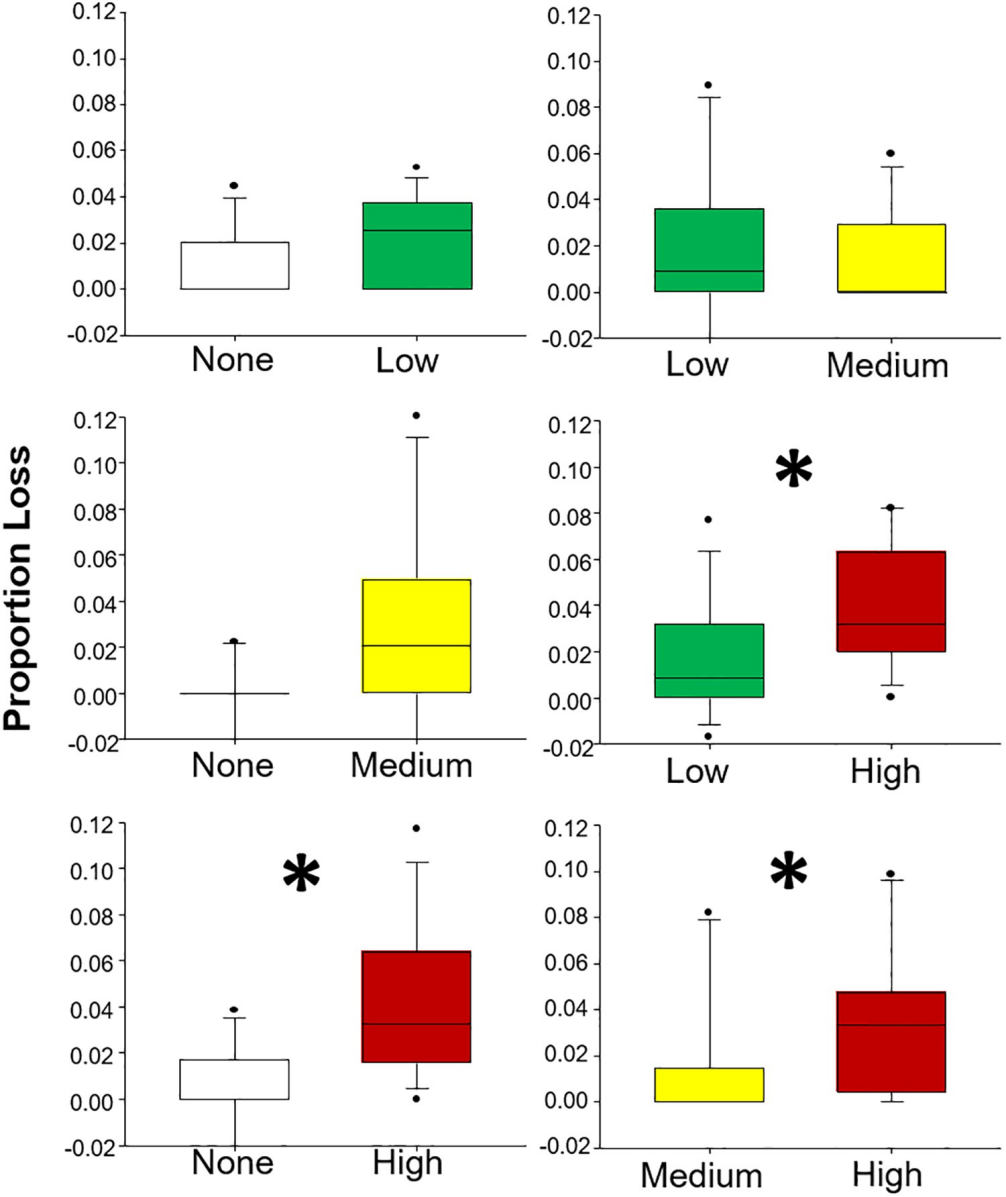
Results from Experiment 1: Amphipods. Paired choice experiments using amphipod (*G*. *mucronatus*) herbivores and plants grown in 4 oiled conditions: no (white), low (green), medium (yellow), and high (red). Asterisk indicates that a statistically significant difference exists.

Amphipods also showed similar trends in consumption preferences, albeit with less amount of material consumed than grass shrimp (generally less than 10% of material consumed). Significant preferences (Table 1, Fig 3) were detected for the following comparisons: None vs High, Low vs High, and Medium vs High. Again, herbivores preferred plant tissue that had been grown under higher oil concentrations.

#### Experiment 2: Foraging rate tests

**Fig 4:**
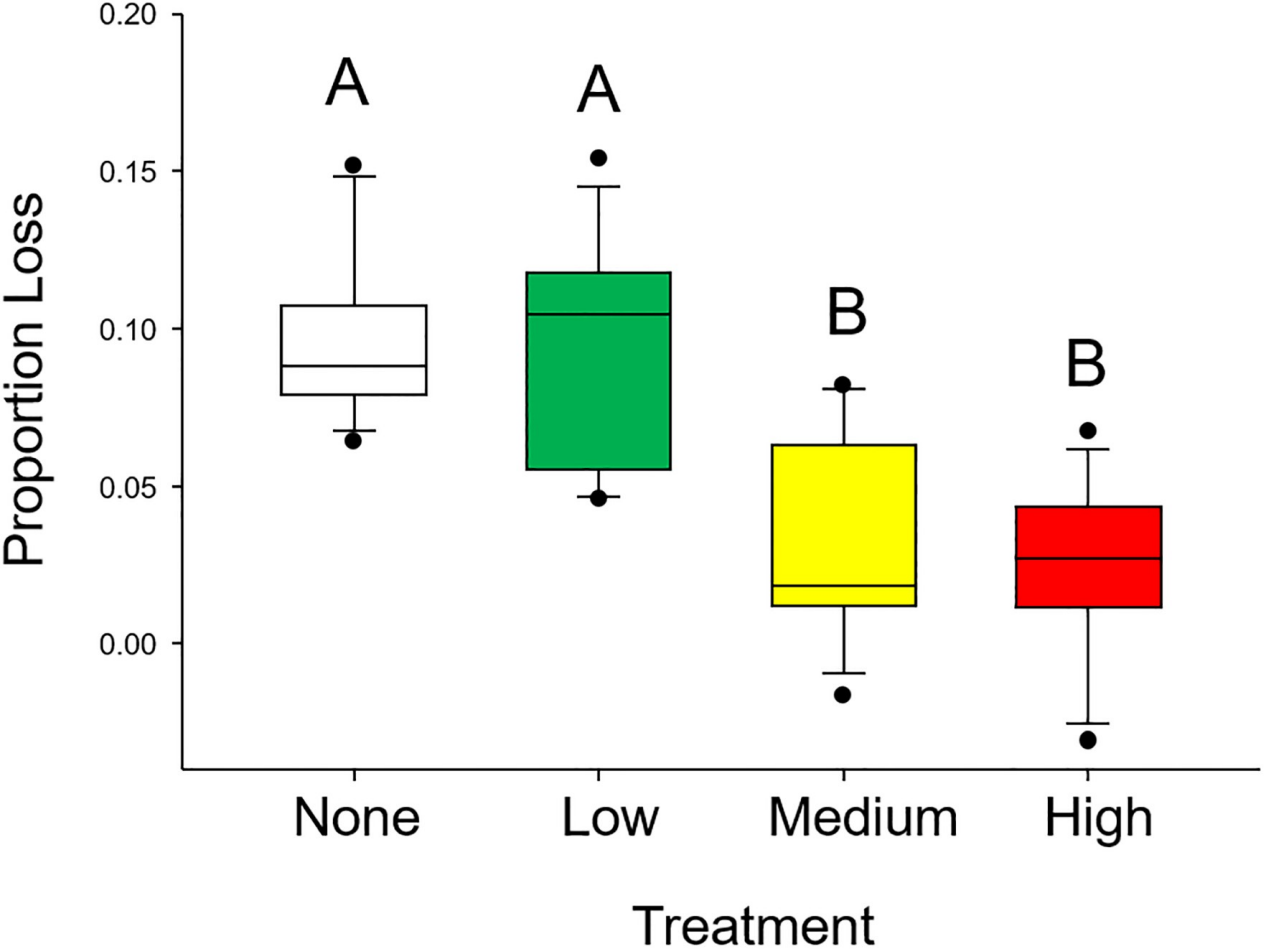
Results from Experiment 2: Grass shrimp. Foraging rate experimental results indicating consumption by grass shrimp (*P*. *pugio*). Colors represent plants grown under one of 4 different oil conditions: no (white), low (green), medium (yellow), and high (red). Different letters indicate statistically significant differences.

When not given a choice between plant tissues, different trends were observed with a larger proportion of tissue consumed in tissues with none/lower oil exposures. For grass shrimp, significant differences were detected in the amount of plant tissue consumed among plants from different oil exposures (F_3,44_ = 20.294, p < 0.001; Fig 4). More plant material was consumed from none (9.6%) and low (9.3%) treatments than from artificial leaves from medium (3.1%) and high (2.3%) oil concentrations.

**Fig 5:**
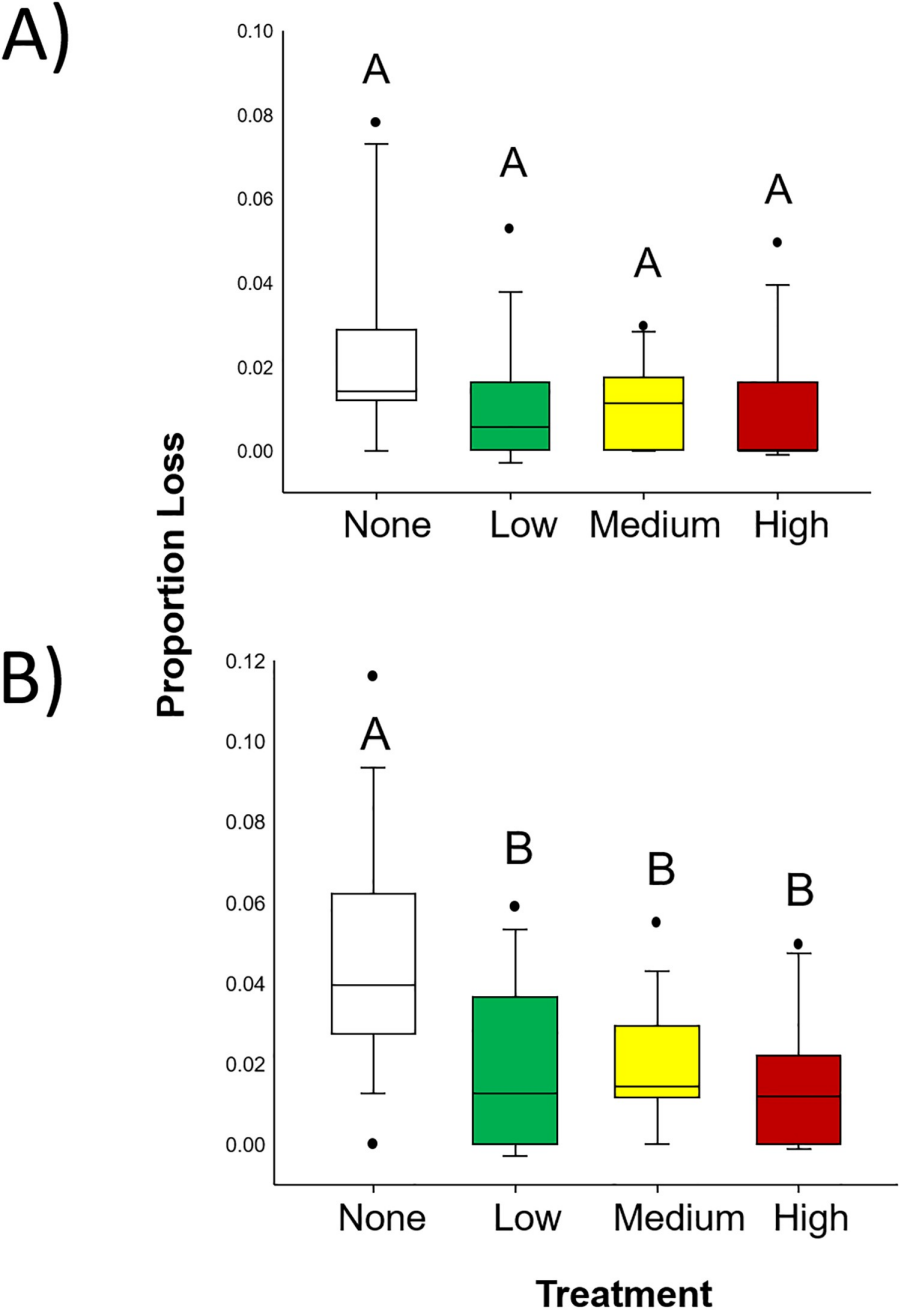
Results from Experiment 2: Amphipods. Foraging rate experimental results indicating consumption by amphipods (*G*. *mucronatus*) over 24 (A) and 48 (B) hour experimental durations. Colors represent plants grown under one of 4 different oil conditions: no (white), low (green), medium (yellow), and high (red). Different letters indicate statistically significant differences.

For amphipods, similar trends were evident. When allowed to forage for 24 hours, no difference was detected (F_3,68_ = 2.51, p = 0.066, Fig 5A). However, amphipods consumed very little tissue during these trials (2.3% in no oil, and around 1% in other treatments). After a longer time to forage and almost twice the amount of plant material consumed, significant differences were detected (F_3,68_ = 7.14, p < 0.001; Fig 5B). In these 48 hours trials, significantly more plant material was consumed from the no oil treatment (4.4%) than from plants grown in low (1.8%), medium (2.0%), or high (1.5%) oil treatments.

## Discussion

Over the past several decades, the GOM has been central to many ecological disturbances including invasive species [33, 34], hurricanes [35], wetland loss/erosion [36], overharvesting/fishery collapse [37], and, most recently, the Deepwater Horizon oil spill. To date, studies conducted in nearshore waters have highlighted the impacts of oil on saltmarshes [8], arthropod insects [38], benthic invertebrates [39, 40], fishes [41–43], birds [44], and marine mammals [45]. While the impact of oil on interactions among organisms is the topic of theoretical [9] and field measurements [46], manipulative experiments quantifying the transfer of energy among organisms have been rare, especially at the primary producer-herbivore trophic level.

Historically, coastal and marine submerged macrophytes were thought to provide little energy to higher trophic levels through herbivory due to high C:N ratios [47–49] and the indigestibility of cellulose content [50]. More recently, however, there is acknowledgement that these plants do play an important role in coastal food webs [51] because: 1) present-day measurements likely represent a very conservative estimate compared to the historical importance of these macrophytes due to the extirpation and global decline of large grazers such as marine mammals, reptiles, and waterfowl [52–54]; 2) nitrogen concentrations in grasses are actually similar to algae [55]; and 3) many herbivorous fishes [56] and widespread abundance of invertebrates mesograzers such as amphipods and urchins [57, 58] exhibit generalist feeding behaviors. While estimates of seagrass production reaching higher trophic levels is highly variable (ranging from ~3–100%, [51, 59]) and site and season dependent, it is now recognized that these plants do play an important role in coastal food webs and, as such, these food webs are at risk from anthropogenic disasters such as oil spills.

Herbivore preference for plants with higher nitrogen content has been well documented in studies of both terrestrial [60–62] and marine herbivores [51]. In marine organisms, a wide range of herbivores have exhibited this foraging pattern, including invertebrates [28, 63], fishes [26, 64], and large herbivores such as turtles [47, 65, 66] and dugongs [67]. Here, we documented a consistent decrease in the C:N ratio of plants as oil exposure increased. This change in plant chemical composition coincided with preferential feeding on oil-exposed plant tissues with higher relative N contents and, when no choice was present, grazers consumed greater proportions of tissues without oil exposure or with lower exposures that had lower relative N contents under laboratory conditions.

One potential explanation for the seemingly contradictory result of preference for higher exposure leaves (higher nitrogen) in choice trials, yet consumption of more tissue of lower exposure (lower nitrogen) is that this represents a compensatory response for grazers [28]. In short, grazers may need to consume more plant tissue when the food has lower nitrogen content to meet metabolic demands. This is a trend that has been previously observed in seagrass ecosystems. Valentine and Heck [28] performed a series of field and lab experiments manipulating nutrients (and therefore leaf nitrogen content) in *Thalassia testudinum* and found that *Lytechinus variegatus* urchins fed at higher rates from low nitrogen and consumed less from nitrogen-enriched leaves such that approximately equivalent amounts of nitrogen were consumed regardless of mass of tissue that needed to be ingested. This study confirms that these findings are also true for *Ruppia* and shrimp/amphipod herbivores and agrees with their finding [28] and adds that PAH contamination may not be a deterrent for grazers, especially when herbivores can benefit from getting high N tissues.

Based on the results of this study, we hypothesize that significant food web impacts may occur as a result of sublethal oil exposure to plants that form a foraging base for the food web. Despite this, we also acknowledge a number of drawbacks that limit the findings of this study and provide fruitful topics for future research. The study conducted here was a lab-based assessment, and future efforts need to verify these patterns in the field and confirm these patterns in a more realistic setting where variability in environmental conditions, predator presence, and additional food choices exist. Moreover, while plants were exposed to oil, herbivores were not exposed to oil, which may additionally alter herbivore health and physiology and impact foraging activities. Herbivores may also exhibit long-term consequences from foraging on oil contaminated tissues that were not documented within the time frame of this study. Additional research documenting mortality rates for these herbivores, as well as sublethal implications such as foraging, reproduction, and predator response, could yield greater insights into the cumulative impacts of oil in coastal environments. Changes in feeding could be due to contaminant loads in tissue, which were not measured during this study. By switching between foraging on unpolluted (lower N) and oil-contaminated tissues (higher N), herbivores may be able to meet metabolic demands while keep contaminant loads in check. While we hypothesized that changes in choice patterns and feeding rates were the result of C:N ratios, it is also possible that an undocumented and unquantified covarying variable influenced this trend. For example, C:N may be correlated with phenolics or other chemical defenses that would alter feeding patterns [68]. Moreover, we know little about the levels of ecological significance in these ratios and a better understanding of food web dynamics may be attained by determining variability in C:N ratios and the ensuing food web consequences. Finally, we also lack a detailed understanding of the mechanisms behind the reported shift in plant chemical composition. We speculate that shifts in microbial communities may have altered the available resources for the plant and resulted in the shift documented here. Moreover, the change in C:N content could be due to mortality of old leaves and rapid new growth, which may have lower C:N ratios.

In conclusion, research to date has focused primarily on the direct impacts of oil [69, 70]. However, findings presented here indicate that important indirect effects may occur through the subtle alterations of trophic pathways that link primary producers with higher trophic levels. Expanding these lab-based results into field settings is critical for developing a more comprehensive understanding of oil’s direct and indirect impacts in coastal ecosystems. Incorporating such knowledge into ongoing ecological modeling efforts will elicit greater insights into food webs, threats, and resilience of northern GOM estuaries, with the key implications for the management and protection of these vital areas.
